# Identification of a novel gene in *Pseudomonas aeruginosa* promotes persister formation by repressing translation and cell division

**DOI:** 10.1128/aac.01274-25

**Published:** 2026-02-26

**Authors:** Jie Feng, Yifan Bian, Liwen Yin, Congjuan Xu, Zhihui Cheng, Yongxin Jin, Shouguang Jin, Weihui Wu

**Affiliations:** 1 State Key Laboratory of Medicinal Chemical Biology, Key Laboratory of Molecular Microbiology and Technology of the Ministry of Education, Department of Microbiology, College of Life Sciences, Nankai University 117931, Tianjin, China; Entasis, Big Bay, Michigan, USA

**Keywords:** persister, *Pseudomonas aeruginosa*, meropenem, *PA2171*, protein translation, cell division

## Abstract

Bacterial persisters are dormant subpopulations that survive antibiotic killing without genetic change. These cells contribute to recalcitrant infections and serve as reservoirs for emergence of antibiotic resistance mutations. *Pseudomonas aeruginosa* is an opportunistic pathogen that is highly resistant to a variety of antibiotics. To characterize the global gene expression profiles of *P. aeruginosa* persister cells and resuscitating cells, we collected live cells after treatment with a lethal dose of meropenem and performed transcriptomic analysis. The *PA2171* gene was upregulated in the persister cells and downregulated during resuscitation. Overexpression of *PA2171* promoted persister formation. Affinity chromatography revealed that the PA2171 protein binds to ribosome and FtsZ, which are involved in translation and cell division, respectively. By measuring protein synthesis in live cells and an *in vitro* translation system, we demonstrated the direct role of PA2171 in repressing protein translation. Meanwhile, fluorescence microscopy and a light scattering assay demonstrated that PA2171 influences cell division by inhibiting FtsZ polymerization. Overall, our results revealed that PA2171 promotes persister formation by coordinating translational arrest and division blockade.

## INTRODUCTION

Bacterial persisters are a small group of phenotypic variants that survive lethal doses of antibiotics (1–3). Unlike antibiotic-resistant mutants, persister cells display transient tolerance to antibiotics (1). Persister cells are thought to be a major cause of chronic and refractory bacterial infections (4–9), as well as reservoirs for the generation of antibiotic-resistant mutants (10). In addition, mutation rates were found to be increased in persister cells (11). Understanding the molecular mechanisms of bacterial persister formation is required for the development of effective treatment strategies to eradicate these highly antibiotic-tolerant bacterial cells.

Bacterial persister formation is driven by both stochastic and responsive mechanisms (12). Stochastic persister formation is typically interpreted as bet-hedging—that is, an evolutionary strategy relying on phenotypic heterogeneity to maximize the fitness of an isogenic population in dynamic environments (13, 14). Direct observations from single-cell microfluidics and flow cytometry revealed that cells surviving antibiotic treatment are part of a preexisting dormant subpopulation of exponentially growing *Escherichia coli* (15, 16). Furthermore, bacteria can respond to environmental cues by phenotypic conversion into persister cells, such as antibiotics and nutrient starvation (17, 18). The persister formation mechanisms primarily include toxin/anti-toxin (TA) systems, stringent response, SOS response, ATP depletion, and oxidative stress. Toxins in TA systems can inhibit bacterial DNA replication, protein synthesis, and peptidoglycan synthesis; depolarize the membrane; or inhibit cell division, resulting in slow-growing or dormant persister cells (19, 20, 21). Nutrient starvation induces bacterial stringent response, leading to the synthesis of the alarmones guanosine pentaphosphate/tetraphosphate, (p)ppGpp. (p)ppGpp globally inhibits growth-related activities such as ribosomal RNA synthesis (17). The SOS response is activated by DNA damage, during which the RecA protein triggers the self-cleavage of LexA, thereby derepressing a series of genes—including TA systems (22, 23). ATP depletion generally suppresses metabolic activity and may trigger protein aggregation (24). Reactive oxygen species (ROS) induce protein aggregates (aggresomes) with reduced metabolic activity and may cause DNA damage (25, 26). The aforementioned processes usually force bacteria into a dormant state and thereby give rise to persister cells.

*Pseudomonas aeruginosa* is a gram-negative opportunistic pathogenic bacterium that causes acute and chronic infections in immunocompromised patients, especially those with cystic fibrosis (27, 28). In *P. aeruginosa*, six chromosome-encoded TA systems have been identified: HicAB, HigBA, RelBE, ParDE, PfiTA, and PacTA. Toxins HicA, HigB, and RelB degrade mRNAs (29–31), and the toxin ParD disrupts DNA replication (32). The toxin PacT inhibits protein translation by acetylating aminoacyl-tRNAs. In addition, PacT suppresses the transcriptional repressive activity of ferric uptake regulator (Fur) on iron uptake-related genes by directly binding to its DNA-binding domain (33). The PfiTA system is encoded by a prophage, and the toxin PfiT induces cell elongation and increases virion production (34). However, compared to the aforementioned bacteria, there are fewer genes identified to contribute to persister formation in *P. aeruginosa*.

Meropenem is one of the potent β-lactam antibiotics used in the treatment of infections caused by gram-negative bacteria, including *P. aeruginosa*. Through inhibiting penicillin-binding proteins (PBPs), meropenem inhibits bacterial cell wall biosynthesis, resulting in osmotic lysis and bacterial death (35). *P. aeruginosa* develops resistance to meropenem through mutations that reduce influx (mutation in the porin gene *oprD*), increase efflux (such as mutations in the efflux pump repressor genes *mexR* and *nalB*), and cause horizontal acquisition of β-lactamase genes (36–38). However, it remains largely unknown how the meropenem treatment triggers persister formation in *P. aeruginosa*. Understanding the mechanism may provide clues to enhance the efficacy of meropenem.

In our previous study, we performed transcriptomic analysis on *P. aeruginosa* persister cells following treatment with a lethal dose of ciprofloxacin. We identified that genes *PA2285* and *PA2287* are upregulated in persister cells and promote persister formation by inhibiting transcription and cell division (39). This finding motivated us to investigate the similarities and differences in global gene expression and mechanisms underlying persister cell formation in *P. aeruginosa* under lethal doses of different antibiotics.

In this study, we collected live *P. aeruginosa* cells after treatment with meropenem and monitored their regrowth in an antibiotic-free medium. The transcriptomic profiles of persister cells and regrown cells were analyzed. By screening genes upregulated in persister cells and downregulated in regrown cells, we identified a novel gene involved in persister formation and elucidated its functional mechanism.

## MATERIALS AND METHODS

### Bacterial strains and growth conditions

The bacterial strains, plasmids, and primers used in this study are listed in Table S1. *P. aeruginosa* PA14 (ATCC 27853) is a wild-type reference strain. All strains were cultured aerobically in Luria-Bertani (LB) medium (1% tryptone, 0.5% yeast extract, and 1% NaCl, at pH 7.4 ± 0.2) at 37°C with constant shaking at 200 rpm. IPTG (BBI Life Sciences, China) was added to the medium at indicated concentrations.

### Bacteria killing assay

The time-dependent killing assay was performed as previously described (39). Briefly, overnight *P. aeruginosa* cultures were diluted into fresh LB to an OD_600_ of 0.05. The bacteria were cultured until the OD_600_ reached approximately 0.8–1.0. Then, antibiotics at the indicated concentrations were added to the medium. At each indicated time point, an aliquot of the culture was taken out. The bacteria were washed with 0.9% NaCl 3 times, followed by serial dilution and plating on LB agar for CFU counting. All experiments were conducted in biological triplicates. Averages and standard deviations are representatives of the biological repeats.

### Persister cell collection

The wild-type PA14 was grown in LB to an OD_600_ of 0.8–1.0. The bacteria were collected by centrifugation for RNA isolation. For the persister cells, PA14 at an OD_600_ of 0.8–1.0 was treated with meropenem (8 μg/mL) in 100 mL LB for 6 h. The bacteria were collected by centrifugation at 10,000 *g* for 15 min at 4°C. To monitor the growth of the cells, the collected cells were resuspended in fresh LB broth and then cultured at 37°C with agitation. For RNA isolation or propidium iodide (PI) staining, the antibiotic-treated bacteria or those regrown in LB were collected by centrifugation. The bacteria were washed twice with PBS and then resuspended in 0.3 M sucrose, followed by centrifugation at 4°C, 10,000 *g* for 3 min to remove cell debris.

### RNA extraction and quantitative RT-PCR (RT-qPCR)

Total RNA was isolated from the collected bacterial cells with an RNeasy Mini kit (Tiangen Biotech, Beijing, China). The cDNA was synthesized from the total RNA by using the PrimeScript Reverse Transcriptase Mix (TaKaRa, China). All real-time PCR primers are listed in Table S2. SYBR Premix Ex TaqTM II (TaKaRa, China) was used in RT-qPCR. The real-time PCR and data analyses were performed with a CFX Connect Real-Time system (Bio-Rad, USA).

### PI staining

The collected bacterial cells were resuspended in PBS and stained with 0.5 μg/mL PI (Dingguo, China) at room temperature in the dark for 15 min. The cells were then subjected to centrifugation at 10,000 *g* for 10 min and resuspended in 1 mL PBS, followed by observation with a fluorescence microscope (OLYMPUS, Tokyo, Japan) with an emitting laser of 635 nm. At least 3 biological repeat assays of each sample were performed.

### Bacterial growth assay

Strains carrying indicated genes on pMMB67EH or the empty vector were cultured in 100 mL of LB broth supplemented with 1 mM of IPTG at 37°C with shaking at 200 rpm for 12 h. Aliquots were taken every 60 min for the measurement of OD_600_ and enumeration of CFU.

### Affinity chromatography

PA14 carrying the 6× His-tagged PA2171 (PA2171-His), glutathione S-transferase (GST) (GST-His) on pMMB67EH, or the empty vector were grown at 37°C in LB. At the OD_600_ of 0.05, 1 mM IPTG was added to the medium to induce the expression of PA2171-His and GST-His for 7 h. The bacteria were collected by centrifugation and resuspended in the lysis buffer (20 mM Tris, 150 mM NaCl, 3 mM β-mercaptoethanol, 0.5% NP-40, and 20 mM imidazole, at pH 8.0). After sonication, the bacterial lysate was subjected to centrifugation at 10,000 *g* for 15 min. The supernatant was incubated with the Ni-NTA agarose (Qiagen, Germany) at 4°C for 2 h. The bound proteins were eluted with an elution buffer (20 mM Tris, 150 mM NaCl, 3 mM β-mercaptoethanol, 0.5% NP-40, and 250 mM imidazole, at pH 8.0). PA2171-His and associated proteins were separated on SDS-PAGE gel and stained with a Coomassie blue staining solution (0.25 g Coomassie brilliant blue R-250, 45 mL methanol, 10 mL glacial acetic acid, and 45 mL distilled water). Protein bands different from the control sample (empty vector and GST-His) were indicated with sequential numbers. The bands were excised from the protein gel, followed by LC-MS/MS analyses.

Wild-type PA14 carrying pPUCP24-*rplL*-His, pPUCP24-*ftsZ*-His, pMMB67EH-PA2171-FLAG, or pMMB67EH was grown to an OD_600_ of 0.6 and incubated with 1 mM IPTG for 6 h. The bacteria were lysed and subjected to chromatography with Ni-NTA beads. The 6× His-tagged RplL/FtsZ and FLAG-tagged PA2171 were detected by Western blot. For Western blots, the anti-6× His tag and Flag tag antibodies were purchased from Milipore (Germany) and Sigma (USA), respectively. The secondary antibodies were purchased from Promega (USA).

### Measurement of protein synthesis in live bacteria

Newly synthesized proteins were determined with the BeyoClick L-homopropargylglycine (HPG) Protein Synthesis Kit with AF488 (BeyoClick HPG-488 Nascent Protein Assay Kit). This kit is based on the incorporation of the methionine analog (HPG) during protein synthesis. Then, the incorporated HPG is labeled with biotin through a click reaction, which enables the detection of newly synthesized proteins by horseradish peroxidase (HRP)-conjugated streptavidin. Briefly, overnight bacterial cultures were diluted into fresh LB to the OD_600_ of 0.05 and cultured until the OD_600_ reached approximately 0.8–1.0. The bacteria were collected by centrifugation and resuspended in the M9 minimal medium containing pre-warmed 1× HPG working solution and 1 mM IPTG. The bacteria were grown at 37°C. At the indicated time points, the bacteria were washed with PBS and lysed by sonication in PBS buffer. After centrifugation at 14,000 *g* for 5 min at 4°C, 50 μL of the supernatant was mixed with 150 μL of the Click reaction solution, followed by incubation at room temperature for 20 min to label the newly synthesized proteins with biotin. The samples were subjected to Western blot analysis with horseradish peroxidase-conjugated streptavidin (Streptavidin-HRP) and enhanced chemiluminescence (ECL). The bacterial total proteins were analyzed by SDS-PAGE and used as the loading control.

### Purification of 6× His-tagged FtsZ and PA2171 (FtsZ-His and PA2171-His)

PA14 carrying pMMB67EH-*ftsZ*-His was grown at 37°C to an OD_600_ of 0.6, followed by culture with 1 mM IPTG for 6 h. The cells were harvested by centrifugation at 4°C and washed once with 20 mM Tris–HCl (pH 7.9). The cell pellets were stored at −80°C. The frozen pellet was thawed on ice and resuspended in a FtsZ anion exchange lysis buffer (low salt) (50 mM Tris, 50 mM KCl, 1 mM EGTA, and 10% glycerol, at pH 8.5) (40, 41). The cells were lysed by passage through a Nano Homogenize Machine (ATS Engineering Inc, China). The lysate was centrifuged at 10,000 *g* for 15 min, and the supernatant was precipitated with 30% ammonium sulfate buffer. The pellet was resuspended with an anion exchange lysis buffer (low salt), and the FtsZ-His was purified using an Ni-NTA column (Qiagen) and dialyzed overnight in 1 L of an FtsZ dialysis buffer (50 mM HEPES, 50 mM KCl, 2.5 mM MgCl2, 1 mM EGTA, and 10% glycerol, at pH 7.5) (40). The proteins were aliquoted and stored frozen at –80°C.

For the purification of PA2171-His, PA14 carrying pMMB67EH-PA2171-His was grown to an OD_600_ of 0.6 and grown in the presence of 1 mM IPTG for 7 h at 37°C. The bacteria were harvested by centrifugation at 4°C and washed once with 20 mM Tris–HCl (pH 7.9). The cell pellets were stored at −80°C. The frozen pellet was thawed in a lysis buffer (20 mM Tris–HCl, 150 mM NaCl, 3 mM β-mercaptoethanol, 0.5% NP-40, and 20 mM imidazole, at pH 8.0) and lysed by sonication. The lysate was subjected to centrifugation at 10,000 *g* for 10 min.

### Measurement of protein synthesis *in vitro*

*In vitro* protein translation was performed using the Promega S30 T7 High-Yield Protein Expression System, which contains the T7 RNA polymerase for transcription and all the necessary components for translation. We utilized the pET41a-GST plasmid to translate the target protein GST. One microgram of the pET41a-GST plasmid and purified PA2171-His or GFP-His (0.1 μg or 1 μg) was mixed with 50 μL of the Protein Expression System on ice in a DNase- and RNase-free 1.5 mL microcentrifuge tube, followed by incubation at 37°C with shaking for 1–3 h. The reaction was stopped by placement in an ice-water bath for 5 min. Then, the yield of the 6× His-tagged GST was determined by Western blotting.

### Determination of cell length and FtsZ ring formation

Cell length was measured as the distance between cell poles. The cell length/FtsZ ring (L/R) ratio was calculated as the total cell length divided by the number of FtsZ rings in the population (40, 41). Significance of length distributions was established employing a chi-square analysis with a significance (a) ≤ 0.001. The significance of FtsZ ring frequencies and L/R ratios was determined using analysis of variance (ANOVA) with ≤ 0.001.

### The 90° angle light-scattering assay to determine FtsZ polymerization

The 90° angle light-scattering assay was performed as previously described (40, 41). Purified FtsZ-His, GST-His, and PA2171-His were diluted to 5 mM in a polymerizing buffer (50 mM MES, pH 6.5, 50 mM KCl, 2.5 mM MgCl_2_, and 1 mM EGTA). FtsZ alone or together with GST-His or PA2171-His (at a 1:1 molar ratio) was mixed with supplementation with 1 mM GTP in 100 µL of the polymerizing buffer. Readings were taken at 310 nm every 0.5 s at 30°C using a Varioskan Flash (Thermo Scientific, USA).

## RESULTS

### Transcriptome analysis of *P. aeruginosa* persister cells after meropenem treatment

**Fig 1:**
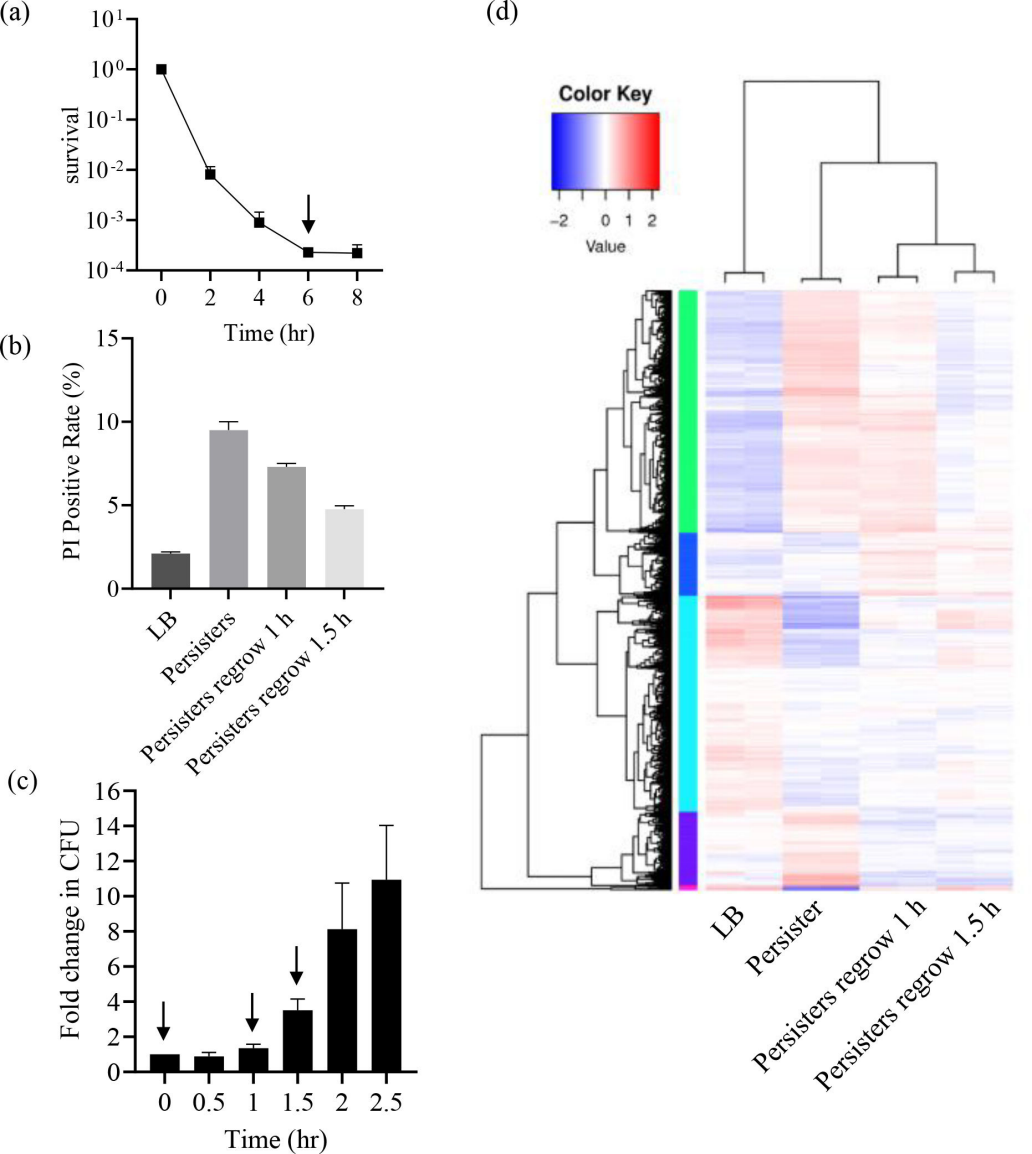
Collection and transcriptome analysis of *P. aeruginosa* persister cells. (**a**) Killing kinetics of the wild-type PA14. (**b**) The cells were stained with propidium iodide (PI) and examined by microscopy. Data shown represent at least two independent experiments with similar results. (**c**) Growth of persister cells. Persister cells were grown in fresh Luria-Bertani (LB). At indicated time points, the numbers of live bacteria were determined by plating. Samples collected for RNA-seq analysis were indicated by arrows. (**d**) Clustering map of differential genes. Clustering was performed based on log_10_ (FPKM + 1) values, where red and blue indicate up- and down-regulated genes, respectively.

To investigate global gene expression profiles of persister cells, we performed bacteria killing assays with cell lysing meropenem. The minimal inhibitory concentration (MIC) of meropenem for wild-type PA14 is 0.5 μg/mL. Treatment of the exponential growth phase PA14 cells with 8 μg/mL meropenem (the clinical resistant breakpoint) resulted in a typical biphasic killing curve, with a rapid decline of live cells in the first 4 h, followed by a slow reduction of live cells (Fig. 1a). To isolate the persister cells, the bacteria were collected at 6 h after meropenem treatment (indicated by an arrow in Fig. 1a). In the collected cells, less than 10% were PI-positive, indicating the majority of the cells were alive (Fig. 1b; Fig. S1). We then monitored the growth of these collected persister cells in fresh LB. Within the first hour, the number of live bacteria remained unchanged. Then, the cell number started to increase, and after 1.5 h, the cells entered a fast growth stage (Fig. 1c). PI staining revealed decreasing percentages of dead cells in the population at the two time points (1 h and 1.5 h) (Fig. 1b; Fig. S1). The collected persister cells and the cells grown in fresh LB for 1 h and 1.5 h were subjected to RNA-seq analyses (Fig. 1d; Fig. S2). Our hypothesis is that genes promoting persister formation might be upregulated in persister cells while downregulated in regrowing cells. A total of 65 genes fitted this criterion (Table S3), among which we chose the top 10 upregulated genes in the persister cells to study their roles in persister formation (Table S4).

### PA2171 promotes persister formation in *P. aeruginosa*

**Fig 2:**
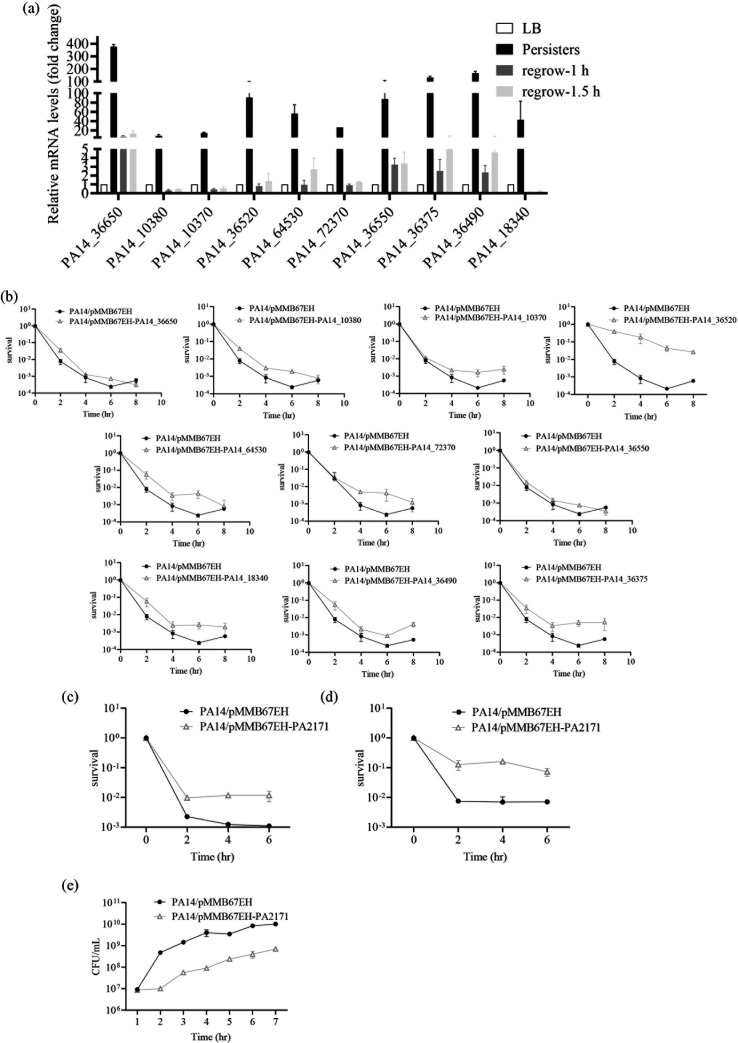
PA2171 contributes to persister formation. (**a**) Expression of the indicated genes was determined by RT-qPCR. (**b**) The survival curves of wild-type PA14 overexpressing indicated genes following meropenem (8 μg/mL) treatment. (**c–d**) The survival curves of wild-type PA14 overexpressing *PA2171* following ciprofloxacin (0.25 μg/mL) (**c**) and tobramycin (4 μg/mL) (**d**) treatment. (**e**) Growth curves of the *PA2171* overexpressing strain and the empty vector control strain.

First, RT-qPCR assays confirmed the upregulation of the 10 genes in persister cells and declined expression in regrowing cells (Fig. 2a). To examine whether these genes contribute to persister formation, we overexpressed each of them in wild-type PA14 and determined bacterial survival following meropenem treatment. Overexpression of all of the tested genes enhanced persister formation (Fig. 2b). Among them, overexpression of *PA14_36520* (*PA2171* in PAO1) showed the most significant effect by increasing bacterial survival by more than 100-fold after meropenem treatment. In addition, overexpression of *PA2171* increased bacterial survival by more than 10-fold after ciprofloxacin and tobramycin treatment (Fig. 2c and d). Meanwhile, overexpression of *PA2171* reduced the bacterial growth rate (Fig. 2e). However, deletion of *PA2171* did not affect bacterial survival following meropenem treatment (Fig. S3), presumably due to redundancy of other persister formation mechanisms, such as *PA2285*, *PA2287,* and multiple TA systems. Collectively, these results demonstrate that PA2171 regulates bacterial growth and persister formation.

### Identification of PA2171-binding proteins

**Fig 3:**
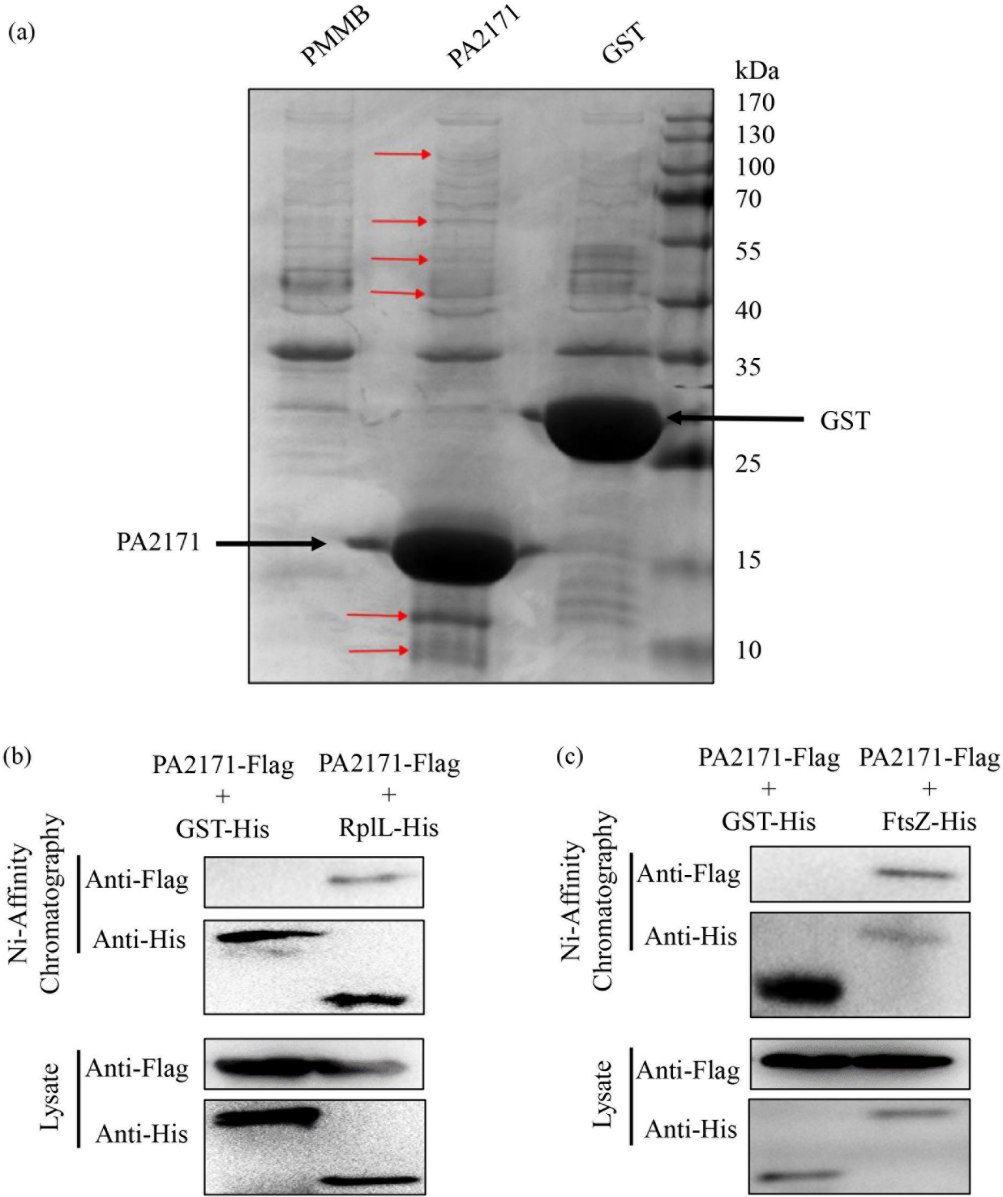
Identification of PA2171-binding proteins. (**a**) PA14 containing pMMB, pMMB-*PA2171*-His, or pMMB-*gst*-His was grown at 37°C in Luria-Bertani (LB). At an OD_600_ of 0.05, 1 mM IPTG was added to the medium. When the OD_600_ reached 0.8–1.0, proteins from bacterial lysates were purified with Ni-NTA beads. PA2171-His and the associated proteins were separated on SDS-PAGE and stained with Coomassie blue staining solution. Protein bands different from the control sample (empty vector and pMMB-*gst*-His) were indicated with arrows. (**b and c**) PA14 carrying *PA217*1-FLAG with pUCP24-*rplL*-His (**b**), pUCP24-*ftsZ*-His (**c**), or the empty vector pUCP24 was grown to an OD_600_ of 0.1 and cultured with 1 mM IPTG for 7 h. The bacterial lysates were subjected to chromatography with Ni-NTA beads. The FLAG-tagged PA2171 and 6× His-tagged RplL or FtsZ were detected by Western blot.

PA2171 is a hemoglobin-like protein (HLP) that consists of five α-helices. HLPs have been found in a wide range of organisms and play roles in oxygen binding, iron sequestration, and chemotaxis (42–46). Based on the structure of PA2171 predicted by AlphaFold 3 (Fig. S4), we speculated that PA2171 lacks DNA- or RNA-binding domains and shows no potential nuclease/protease activity, suggesting that its function may be exerted through protein-protein interactions. To identify its binding targets, we overexpressed a 6× His-tagged PA2171 (PA2171-His) in wild-type PA14 and performed affinity chromatography purification. Proteins co-purified with PA2171-His were separated by SDS-PAGE, and the protein bands distinctive from the control sample were subjected to LC-MS/MS analyses (Fig. 3a). The potential PA2171-interacting proteins are listed in Table S5. Among the proteins, there were 25 ribosomal proteins and the cell division protein FstZ, which play critical roles in bacterial growth. To verify the protein interactions, we co-expressed a C-terminal FLAG-tagged PA2171 (PA2171-FLAG) with a C-terminal 6× His-tagged RplL or FtsZ (RplL-His or FtsZ-His). Since each ribosome contains 4 molecules of the RplL subunits, the RplL-His fusion protein has been used to purify ribosomes (47). Ni-affinity chromatography assays demonstrated the interaction between PA2171-FLAG and RplL-His or FtsZ-His (Fig. 3b and c), indicating the potential role of PA2171 in interfering with translation and cell division.

### PA2171 inhibits protein translation in *P. aeruginosa*

**Fig 4:**
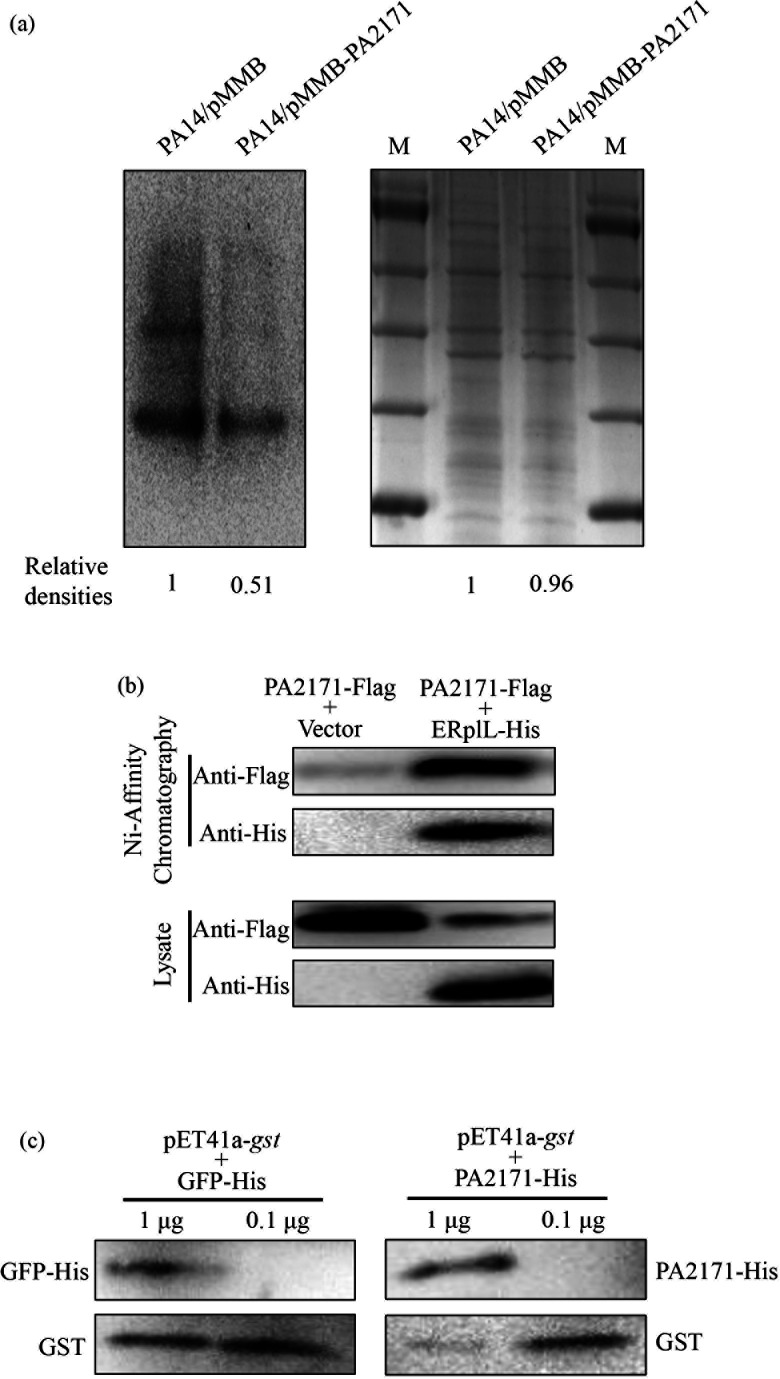
PA2171 directly affects translation. (**a**) PA2171 inhibits protein translation in *P. aeruginosa*. PA14 containing the *PA2171* overexpressing plasmid or the empty vector was grown in the M9 minimal medium containing HPG and 1 mM IPTG. After 4 h, the protein synthesis was determined by measuring HPG incorporation. The bacteria were lysed, and the incorporated HPG is labeled with biotin through a click reaction. Then, the labeled proteins were analyzed by Western blot with Streptavidin-HRP (left panel). The total bacterial proteins were determined by SDS-PAGE and Coomassie brilliant blue staining, which was used as the internal control (right panel). The band densities were determined by ImageJ. M, protein marker. Data represent the results from three independent experiments. (**b**) PA2171 binds to the *E. coli* ribosome. *E. coli* carrying *PA2171*-FLAG with pUCP24-E*rplL*-His or the empty vector pUCP24 was grown to an OD_600_ of 0.1 and cultured with 1 mM IPTG for 7 h. The bacterial lysates were subjected to chromatography with Ni-NTA beads. The FLAG-tagged PA2171 and 6× His-tagged ERplL were detected by Western blot. (**c**) PA2171 decreases the protein yield of the *in vitro* translation system. The pET41a-*gst* plasmid (1 μg) and the purified PA2171-His or GFP-His protein (0.1 or 1 μg) were mixed with 50 μL of the protein expression system, followed by incubation at 37°C for 1 h. Then, the yield of the 6× His-tagged GST was determined by Western blotting.

To verify the role of PA2171 in inhibiting translation, we utilized a BeyoClick HPG Protein Synthesis Kit. In this experiment, IPTG was added to induce the expression of *PA2171*. Simultaneously, HPG was added to the medium, which was incorporated into newly synthesized proteins. Thus, by measuring HPG-labeled proteins, we could determine the impact of PA2171 on translation in live bacteria. Overexpression of *PA2171* repressed protein synthesis (Fig. 4a). Since overexpression of *PA2171* inhibited bacterial growth, we utilized an i*n vitro* translation system to verify that the PA2171-mediated reduction of protein synthesis is through functional inhibition of ribosomes.

The *in vitro* translation system (S30 T7 High-Yield Protein Expression System) is an *E. coli* extract-based cell-free protein synthesis system. We thus first verified the binding of PA2171 to the *E. coli* ribosome by using a C-terminal 6× His-tagged *E. coli* RplL (ERplL-His). As shown in Fig. 4b, the PA2171-FLAG was co-purified with the ERplL-His from *E. coli*, demonstrating an interaction between PA2171 and the *E. coli* ribosome (ERplL-His). Then, we used a T7 promoter-driven C-terminal 6× His-tagged *gst* (*gst*-His) to examine protein synthesis. Addition of 1 μg purified PA2171-His, but not GFP-His, diminished the yield of the GST-His protein (Fig. 4c). Collectively, these results demonstrate a role of PA2171 in inhibiting protein translation.

### PA2171 interferes with cell division by interfering with FtsZ polymerization

**Fig 5:**
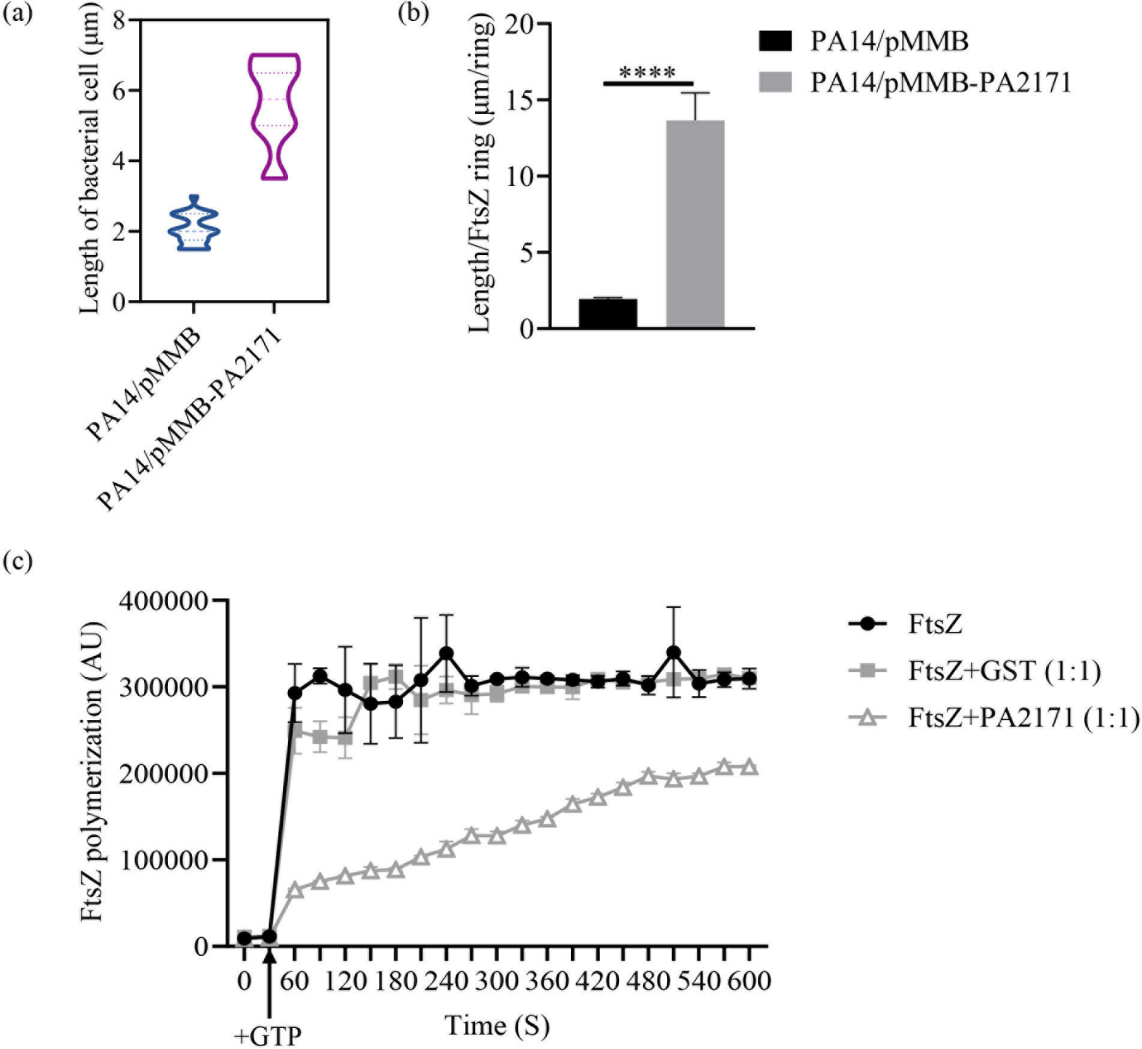
PA2171 inhibits cell division by interacting with the cell division protein FtsZ. (**a**) Cell lengths of PA14 strains overexpressing *PA2171* or carrying the empty vector are shown using violin plots, with 50 cells measured for each strain. (**b**) The length/ring formation (L/R) ratio in PA14/pMMB and PA14/pMMB-*PA2171*. (**c**) FtsZ polymerization in the presence of GST or PA2171 was determined with the 90° angle light-scattering assay. ****, *P* < 0.0001, unpaired t-test.

Next, we investigated the impact of PA2171 on cell division by measuring cell length, FtsZ ring formation, and the cell length/ring formation (L/R) ratio that was used to calculate the ability of mature septum formation and cell division (40, 41). Overexpression of *PA2171* increased the average cell length by 2.3-fold compared to the empty vector group (Fig. 5a; Fig. S5 and S6). By using an *ftsZ-gfp* fusion gene, we found that overexpression of *PA2171* reduced the FtsZ ring formation by 4.5-fold (Fig. S6) and increased the L/R ratio by 7-fold (Fig. 5b).

To verify the role of PA2171 in interfering with FtsZ ring formation, we measured FtsZ polymerization *in vitro* by using the 90° angle light-scattering assay (40, 41). Addition of 1 mM GTP to 5 mM purified FtsZ led to a rapid increase in light scattering, demonstrating polymerization of FtsZ. Addition of purified PA2171, but not GST, delayed FtsZ polymerization (Fig. 5c). In combination, these results indicate that PA2171 interferes with cell division by interacting with the cell division protein FtsZ.

## DISCUSSION

Bacterial persisters are dormant cells that survive antibiotics without genetic change, causing chronic and relapsed infections (48, 49). Understanding the mechanisms of persister formation is pivotal for overcoming the limitations of conventional antibiotic therapies. Here, in this study, we examined global gene expression of *P. aeruginosa* persister cells after meropenem treatment. Although washing with sucrose eliminated most lysed cells, the RNA-seq samples may still contain RNA from nonpersister cells. In the collected cells, less than 10% cells were PI-positive (Fig. 1b; Fig. S1). However, we cannot exclude the possibility that there were viable but nonculturable (VBNC) cells. The physiological state and transcriptomic profile of these cells are different from persister cells. The presence of VBNC cells will affect the overall RNA-seq results. Therefore, it is critical to examine the roles of differentially expressed genes in the RNA-seq results. Our results demonstrated that PA2171 inhibits protein translation and cell division by directly targeting ribosome and FtsZ, respectively, which promotes persister formation. Overexpression of *PA2171* increased bacterial survival by 100-, 10-, and 10-fold following meropenem, ciprofloxacin, and tobramycin treatment, respectively, revealing different persister levels for each of the antibiotics. Further studies are warranted to understand the different effects against the antibiotics by PA2171.

Besides *PA2171*, our transcriptomic analysis revealed upregulation of 1,567 genes in the persister cells. Among those genes, there are multiple operons composed of two genes, such as *PA14_72360–PA14_72370*, *PA14_36540–PA14_36550*, *PA14_36375–PA14_36390*, *PA14_27650–PA14_27660*, *PA14_06080*–PA14_*06090*, *PA14_47120–PA14_47130*, *PA14_62680–PA14_62690*, *PA14_24740–PA14_24770*, and *PA144_35010–PA14_35060*. Their roles in persister cell formation remain to be further investigated. Meanwhile, 463 genes were downregulated in the persister cells and upregulated during regrowth. Among those, there might be genes repressing persister formation or promoting bacterial regrowth. Understanding the functions of these genes might provide targets to interfere with persister formation or resuscitation.

In our previous study on *P. aeruginosa* persister cells that survived ciprofloxacin treatment, we focused on the operon *PA14_35060–PA14_35010* (*PA2282–PA2287*) (39). PA2285 and PA2287 contribute to persister formation by binding to RpoC and FtsZ and inhibiting their functions. In our previous transcriptomic analysis, *PA2171* was also upregulated, although not as much as the operon *PA2282–PA2287*. Here, we found that overexpression of *PA2171* increased bacterial survival by 10-fold following ciprofloxacin treatment, whereas overexpression of *PA2285* or *PA2287* enhanced the survival by 100-fold (39). There is an essential difference in the bactericidal mechanisms between meropenem and ciprofloxacin. Meropenem mainly inhibits the cross-linking of peptidoglycans, leading to defects in the cell wall structure and ultimately cell lysis (35). Ciprofloxacin targets bacterial DNA gyrase, which results in DNA breakage and replication arrest (50, 51). These results indicate different roles of those genes in bacterial survival under treatment of antibiotics with different bactericidal mechanisms.

In recent years, there has been extensive research on TA systems that promote persister formation. Multiple TA systems have been found to induce dormancy by inhibiting protein synthesis (52–54). Among the currently identified six *P. aeruginosa* chromosome-encoded type II TA systems, three toxins inhibit translation. Toxin RelE is a global inhibitor of translation that is activated by nutritional stress. RelE-mediated cleavage of mRNAs depends on translation of the mRNAs (29). Toxin HigB belongs to the RelE family. As an endoribonuclease, HigB binds to ribosomes and cleaves within mRNA-coding regions at AAA triplet sequences (31, 55, 56). Toxin HicA induces cleavage of mRNAs and the tmRNA by a ribosome-independent manner, concomitantly reducing the global rate of translation (30). The *PA2171* gene is localized in the *PA2171–PA2173* operon. PA2172 contains an aminopeptidase domain, while PA2173 has no predicted functional domain (57). Further studies are needed to investigate whether PA2171 is a toxin of the typical TA system.

This study demonstrated that PA2171 promotes persister formation by repressing protein translation and inhibiting FtsZ polymerization. We previously found that PA2285 and PA2287 also bind to FtsZ and inhibit its polymerization (39). There are also reports demonstrating that several factors promote cell dormancy through FtsZ. Toxin HigB degrades the *ftsZ* mRNA, arresting cells in a pre-division state, which can survive exposure to antibiotics (58, 59). In biofilms, sublethal doses of antibiotics induce ROS-mediated DNA damage, which leads to activation of the SOS response and subsequent upregulation of *sulA*. SulA binds to FtsZ and prevents divisome formation (60, 61). These studies collectively reveal multiple mechanisms that inhibit cell division by targeting FtsZ, which promotes persister formation in *P. aeruginosa*.

In this study, we demonstrated that PA2171 is upregulated in persister cells and promotes *P. aeruginosa* persister formation. However, the regulatory mechanism of *PA2171* remains to be elucidated. Meropenem inhibits peptidoglycan synthesis, which activates the regulator AmpR and subsequent upregulation of the chromosomal β–lactamase gene *ampC* in *P. aeruginosa* (62, 63). Combining RNA-seq and chromatin immunoprecipitation (ChIP)-Seq, a previous study revealed genes regulated by AmpR (64). However, the *PA2171* operon was not found in the AmpR regulon (64). It has been demonstrated that bactericidal antibiotics induce reactive oxygen production, which activates oxidative stress, SOS, and stringent responses, as well as the sigma factor RpoS (15, 17, 18, 22, 23, 25, 26, 65, 66). Our RNA-seq results revealed upregulation of the catalase genes *katA* and *katB*, alkyl hydroperoxide reductase subunit genes *ahpF* and *ahpC,* and superoxide dismutase genes *sodA* and *sodB*, which might contribute to bacterial survival. However, genes involved in DNA repair (*recN*, *recF*, *uvrC*, and *uvrD*) or ppGpp metabolism (*relA* and *spoT*) were not upregulated (Table S6). These results demonstrate that the oxidative stress response and RpoS were activated in the collected persister cells. We are taking efforts to investigate whether these pathways are involved in the regulation of *PA2171*. In addition, DNA pull-down assay using the *PA2171* promoter may reveal its regulator. Overall, our study revealed that PA2171 promotes persister formation through two key mechanisms, inhibiting protein translation and cell division. Our results shed light on the complex gene expression profile and formation mechanisms of persister cells in *P. aeruginosa*.

## Data Availability

RNA-seq data have been deposited in the NCBI Sequence Read Archive (SRA) with accession code PRJNA1298949.

## SUPPLEMENTAL MATERIAL

Supplemental material aac.01274-25-s0001.docx Tables S1 to S6; Fig. S1 to S6.

ASM does not own the copyrights to Supplemental Material that may be linked to, or accessed through, an article. The authors have granted ASM a non-exclusive, world-wide license to publish the Supplemental Material files. Please contact the corresponding author directly for reuse.
