# Supplementary material for: Identification of a novel gene in Pseudomonas aeruginosa promotes persister formation by repressing translation and cell division
Source: Antimicrob Agents Chemother. 2026 Feb 26;70(4):e01274-25. doi: 10.1128/aac.01274-25 (PMC13041383; doi:10.1128/aac.01274-25)
Supplement: Supplemental material — Tables S1 to S6; Fig. S1 to S6. [file aac.01274-25-s0001.docx]

**Table S1 Bacteria and plasmids used in this study.**

| Bacteria | Description | Source |
| --- | --- | --- |
| PA14 | Wild type PA14 | Lab stock |
| PA14/pMMB67EH | PA14 with pMMB67EH empty vector | This study |
| PA14/pMMB67EH-PA14_36650 | A14 with pMMB67EH- *PA14_36650* | This study |
| PA14/pMMB67EH-PA14_10380 | A14 with pMMB67EH- *PA14_10380* | This study |
| PA14/pMMB67EH-PA14_10370 | A14 with pMMB67EH- *PA14_10370* | This study |
| PA14/pMMB67EH-PA14_36520(PA2171) | A14 with pMMB67EH- *PA14_36520* | This study |
| PA14/pMMB67EH-PA14_65430 | A14 with pMMB67EH- *PA14_65430* | This study |
| PA14/pMMB67EH-PA14_72370 | A14 with pMMB67EH- *PA14_72370* | This study |
| PA14/pMMB67EH-PA14_36550 | A14 with pMMB67EH- *PA14_36550* | This study |
| PA14/pMMB67EH-PA14_18340 | A14 with pMMB67EH- *PA14_18340* | This study |
| PA14/pMMB67EH-PA14_36490 | A14 with pMMB67EH- *PA14_36490* | This study |
| PA14/pMMB67EH-PA14_36375 | A14 with pMMB67EH- *PA14_36375* | This study |
| PA14/pMMB67EH- PA14_36520(PA2171)-His | PA14 with pMMB67EH-*PA14_36520*(*PA2171*) carrying the 6×His tag | This study |
| PA14/pMMB67EH-*GST*-His | PA14 with pMMB67EH-*GST* carrying the 6×His tag | This study |
| PA14/pUCP24-*rplL*-His | PA14 with pUCP24-*rplL* carrying the 6×His tag | This study |
| PA14/pUCP24-*ftsZ*-His | PA14 with pUCP24-*ftsZ* carrying the 6×His tag | This study |
| PA14/pMMB67EH- PA14_36520(PA2171)-FLAG | PA14 with pMMB67EH-*PA14_36520*(*PA2171*) carrying the FLAG tag | This study |
| PA14/pMMB67EH- PA14_36520(PA2171)-FLAG/pUCP24-GST-His | PA14 with pMMB67EH-*PA14_36520*(*PA2171*) carrying the FLAG tag and pUCP24-GST-His | This study |
| PA14/pMMB67EH/pUCP24-*rplL*-His | PA14 with pMMB67EH empty vector and pUCP24-*rplL* carrying the 6×His tag | This study |
| PA14/pMMB67EH/pUCP24-*ftsZ*-His | PA14 with pMMB67EH empty vector and pUCP24-*ftsZ* carrying the 6×His tag | This study |
| PA14/pMMB67EH- PA14_36520(PA2171)-FLAG/pUCP24-*rplL*-His | PA14 with pMMB67EH-*PA14_36520*(*PA2171*)carrying the FLAG tag and pUCP24-*rplL* carrying the 6×His tag | This study |
| PA14/pMMB67EH- PA14_36520(PA2171)-FLAG/pUCP24-*ftsZ*-His | PA14 with pMMB67EH-*PA14_36520*(*PA2171*) carrying the FLAG tag and pUCP24-*ftsZ* carrying the 6×His tag | This study |
| PA14/pMMB67EH-*ftsZ*-His | PA14 with pMMB67EH-*ftsZ* carrying the 6×His tag | This study |
| DH5α/pMMB67EH | DH5α with pMMB67EH empty vector |  |
| DH5α/pMMB67EH- PA14_36520(PA2171)-FLAG | DH5α with pMMB67EH-*PA14_36520*(*PA2171*) carrying the FLAG tag | This study |
| DH5α/pUCP24 | DH5α with pUCP24-GST-His | This study |
| DH5α/pUCP24-ERplL-His | DH5α with pUCP24-*ErplL* carrying the 6×His tag | This study |
| DH5α/pMMB67EH- PA14_36520(PA2171)-FLAG/pUCP24 | DH5α with pMMB67EH-*PA14_36520*(*PA2171*) carrying the FLAG tag and pUCP24 empty vector | This study |
| DH5α/ pMMB67EH- PA14_36520(PA2171)-FLAG/pUCP24- ERplL-His | DH5α with pMMB67EH-*PA14_36520*(*PA2171*) carrying the FLAG tag and pUCP24-*ErplL* carrying the 6×His tag | This study |
| PA14/pMMB67EH/pUCP24-*ftsZ*-egfp | PA14 with pMMB67EH empty vector and pUCP24-*ftsZ* carrying egfp tag | This study |
| PA14/pMMB67EH- PA14_36520(PA2171)-His/pUCP24-*ftsZ*-egfp | PA14 with pMMB67EH-*PA14_36520*(*PA2171*) carrying the 6×His tag and pUCP24-*ftsZ* carrying egfp tag | This study |
| Plasmids |  |  |
| pMMB67EH | Expression vector with tac promoter; AmP^r^ | [1] |
| pMMB67EH-Gm | Expression vector with tac promoter; Gm^r^ | Laboratory preservation |
| pUCP24 | Expression vector with *tac* promoter; Gm^r^ | [2] |
| pET41a-GST | Expression vector with T7 promoter driven C-terminal 6×His tagged *gst* (*gst-*His) | Laboratory preservation |
| pMMB67EH-PA14_36650 | *PA14_36650* gene driven by tac promoter on pMMB67EH; Gm^r^ | This study |
| pMMB67EH-PA14_10380 | *PA14_10380* gene driven by tac promoter on pMMB67EH; Gm^r^ | This study |
| pMMB67EH-PA14_10370 | *PA14_10370* gene driven by tac promoter on pMMB67EH; Gm^r^ | This study |
| pMMB67EH-PA14_36520(PA2171) | *PA14_36520*(*PA2171*) gene driven by tac promoter on pMMB67EH; Gm^r^ | This study |
| pMMB67EH-PA14_65430 | *PA14_65430* gene driven by tac promoter on pMMB67EH; Gm^r^ | This study |
| pMMB67EH-PA14_72370 | *PA14_72370* gene driven by tac promoter on pMMB67EH; Gm^r^ | This study |
| pMMB67EH-PA14_36550 | *PA14_36550* gene driven by tac promoter on pMMB67EH; Gm^r^ | This study |
| pMMB67EH-PA14_18340 | *PA14_18340* gene driven by tac promoter on pMMB67EH; Gm^r^ | This study |
| pMMB67EH-PA14_36490 | *PA14_36490* gene driven by tac promoter on pMMB67EH; Gm^r^ | This study |
| pMMB67EH-PA14_36375 | *PA14_36375* gene driven by tac promoter on pMMB67EH; Gm^r^ | This study |
| pMMB67EH- PA14_36520(PA2171)-His | *PA14_36520*(*PA2171*) gene with His-tag driven by tac promoter on pMMB67EH; Gm^r^ | This study |
| pMMB67EH-*GST*-His | GST with His-tag driven by tac promoter on pMMB67EH; Gm^r^ | This study |
| pUCP24-GST-His | GST protein with His-tag driven by lac promoter on pUCP24; Gm^r^ | This study |
| pUCP24-*rplL*-His | *rplL* gene with His-tag driven by lac promoter on pUCP24; Gm^r^ | This study |
| pUCP24-*ftsZ*-His | *ftsZ* gene with His-tag driven by lac promoter on pUCP24; Gm^r^ | This study |
| pMMB67EH- PA14_36520(PA2171)-FLAG | *PA14_36520*(*PA2171*) gene with FLAG-tag driven by tac promoter on pMMB67EH; Amp^r^ | This study |
| pMMB67EH-*ftsZ*-His | *ftsZ* gene with His-tag driven by tac promoter on pMMB67EH; Gm^r^ | This study |
| pUCP24-ERplL-His | *E. coli* *rplL* gene with His-tag driven by lac promoter on pUCP24; Gm^r^ | This study |
| pUCP24-*ftsZ*-egfp | *ftsZ* gene with egfp-tag driven by lac promoter on pUCP24; Gm^r^ | This study |

[1] Jens P.Fürste, Werner Pansegrau, Ronald Frank et al. Molecular cloning of the plasmid RP4 primase region in a multi-host-range tacP expression vector. *Gene* 1986; 4: 13.

[2] Heurlier K, Williams F, Heeb S, Dormond C, Pessi G, Singer D, Cámara M, Williams P, Haas D. Positive control of swarming, rhamnolipid synthesis, and lipase production by the posttranscriptional RsmA/RsmZ system in *Pseudomonas aeruginosa* PAO1. *J Bacteriol*. 2004 May;186(10):2936-45. doi: 10.1128/JB.186.10.2936-2945.2004.

**Table S2 Primers used in this study.**

| Primer | Sequence 5’-3’ | Function |
| --- | --- | --- |
| PA14_36650-pmmb-F | TTCACACAGGAAACAGAATTCACACAGGAGGACAGC | Over-expression of PA14_36650 gene |
| PA14_36650-pmmb-R | TCCGCCAAAACAGCCAAGCTTTCAATGGTGATGGTGATGATGGTAGTGGGCCTGGGC |  |
| PA14_10380-pmmb-F | TTCACACAGGAAACAGAATTCTGATGAAGGAATAGC | Over-expression of PA14_1038 gene |
| PA14_10380-pmmb-R | TCCGCCAAAACAGCCAAGCTTTCAATGGTGATGGTGATGATGAGGTCCACTCGGTGT |  |
| PA14_10370-pmmb-F | TTCACACAGGAAACAGAATTCATCAGGAGAACCGCC | Over-expression of PA14_10370 gene |
| PA14_10370-pmmb-R | TCCGCCAAAACAGCCAAGCTTTCAATGGTGATGGTGATGATGTGGCATCAGCCGGTC |  |
| PA2171-pmmb-F | TTCACACAGGAAACAGAATTCACAAACCGATGAGGAGCGACC | Over-expression of PA2171 gene |
| PA2171-pmmb-R | TCCGCCAAAACAGCCAAGCTTTCAATGGTGATGGTGATGATGGGCCGCCCGTTTTTC |  |
| PA14_65430-pmmb-F | TTCACACAGGAAACAGAATTCAGGCACTGTCGATAG | Over-expression of PA14_65430 gene |
| PA14_65430-pmmb-R | TCCGCCAAAACAGCCAAGCTTTCAATGGTGATGGTGATGATGGTCGCCCTTGATCTG |  |
| PA14_72370-pmmb-F | TTCACACAGGAAACAGAATTCACAGGAGAAAACATC | Over-expression of PA14_72370 gene |
| PA14_72370-pmmb-R | TCCGCCAAAACAGCCAAGCTTTCAATGGTGATGGTGATGATGGTACCAGTTCGGATC |  |
| PA14_36550-pmmb-F | TTCACACAGGAAACAGAATTCCAGGAGGAGCGGAAC | Over-expression of PA14_36550 gene |
| PA14_36550-pmmb-R | TCCGCCAAAACAGCCAAGCTTTCAATGGTGATGGTGATGATGGAAAGCCGGATGGAT |  |
| PA14_18340-pmmb-F | TTCACACAGGAAACAGAATTCGCGCGAGGCACAGGC | Over-expression of PA14_18340 gene |
| PA14_18340-pmmb-R | TCCGCCAAAACAGCCAAGCTTTCAATGGTGATGGTGATGATGTGGCTGGCGCACTCC |  |
| PA14_36490-pmmb-F | TTCACACAGGAAACAGAATTCCGAAGCAAGGAGAATTCC | Over-expression of PA14_36490 gene |
| PA14_36490-pmmb-R | TCCGCCAAAACAGCCAAGCTTTCAATGGTGATGGTGATGATGCGGCAGGTTCGGCGG |  |
| PA14_36375-pmmb-F | TTCACACAGGAAACAGAATTCTATCACTCGCACCCATAT | Over-expression of PA14_36375 gene |
| PA14_36375-pmmb-R | TCCGCCAAAACAGCCAAGCTTTCAATGGTGATGGTGATGATGTTCAGGCTGGGCATG |  |
| *rplL*-pUCP24-F | AGCTATGACCATGATTACGAATTCCCGTCCCCCAATACAGGAATTAGAGTC | Over-expression of RplL |
| *rplL*-pUCP24-R | AAAACGACGGCCAGTGCCAAGCTTTTAATGGTGATGGTGATGATGCTTGAGCTCGACTTT |  |
| *ftsZ*-pUCP24-F | TATGACCATGATTACGAATTCCAAAACTAGAAAGGAAGGA | Over-expression of FtsZ |
| *ftsZ*-pUCP24-R | ACGACGGCCAGTGCCAAGCTTTCACTTGTCATCGTCGTCCTTGTAGTCATCGGCCTGACGACGCAG |  |
| PA2171-pmmb-FLAG-F | TTCACACAGGAAACAGAATTCACAAACCGATGAGGAGCGACC | Over-expression of PA2171 gene |
| PA2171-pmmb-FLAG-R | TCCGCCAAAACAGCCAAGCTTTCAGATTACAAGGACGACGATGACAAGGGCCGCCCGTTTTTC |  |
| *ftsZ*-pmmb-F | TTCACACAGGAAACAGAATTCCAAAACTAGAAAGGAAGGA | Over-expression of FtsZ |
| *ftsZ*-pmmb-R | TCCGCCAAAACAGCCAAGCTTTCACTTGTCATCGTCGTCCTTGTAGTCATCGGCCTGACGACGCAG |  |
| ERplL-pUCP24- F | TATGACCATGATTACGAATTCGAGCTCTGATATTCAGGAACAATTTAA | Over-expression of *E. coli* RplL |
| ERplL-pUCP24- R | TGTAAAACGACGGCCAGTGCCAAGCTTTTAATGGTGATGGTGATGATG TTTAACTTCAACTTCAGCGCC |  |
| *ftsZ*-pUCP24-egfp-F1 | AACAGCTATGACCATGATTACGAATTCTAGAAAGGAAGGAGAGGGGAA | Over-expression of FtsZ with egfp flag |
| *ftsZ*-pUCP24-egfp-R1 | AAGTTCTTCTCCCTTACCCATATCGGCCTGACGACGCAG |  |
| *ftsZ*-pUCP24-egfp-F2 | CTGCGTCGTCAGGCCGATATGGGTAAGGGAGAAGAACTT |  |
| *ftsZ*-pUCP24-egfp-R2 | TGCCTGCAGGTCGACTCTAGAGGATCCTTATTTGTATAGTTCATCCAT |  |
| Real time PCR primers | Sequence (5’-3’) | |
| PA14_36650-F | TGACCCGCAACGTCCAGG | |
| PA14_36650-R | AGCGGGGTATCCCCGGAC | |
| PA14_10380-F | ATGAAATCGACCAGGTAT | |
| PA14_10380-R | GTGTCGGCCAGTTGATGG | |
| PA14_10370-F | AAGCCGACGCCTTCGTCG | |
| PA14_10370-R | GCCGGAAAGCCTGCCGGC | |
| PA2171-F | ATTGCTGGTCCATGACCA | |
| PA2171-R | AGCTCTTCCTTGCCGCCG | |
| PA14_65430-F | ATCATCGTCAAGCGCGTC | |
| PA14_65430-R | CTGAAACCGATGGGGTCC | |
| PA14_72370-F | TATCACCTTTCTGATCAT | |
| PA14_72370-R | CTAGCCGCGACGGCGTCC | |
| PA14_36550-F | ACTGGTGGTGGCCCATAG | |
| PA14_36550-R | TGCCGACCAGGGTGCGGG | |
| PA14_18340-F | ATCGACGTCGACACCTTC | |
| PA14_18340-R | GCGGCGCGCGAGCGGAGC | |
| PA14_36490-F | ACGGCCAATGGCCTGCCG | |
| PA14_36490-R | CCTTGACATGGCAATGCC | |
| PA14_36375-F | AAACCCACGACAAGGAAC | |
| PA14_36375-R | TCATGGGTGGCGGCCAGG | |

**Table S3: Genes upregulated in persister cells while downregulated in regrowing cells in RNA-seq result.**

| PAO1 ID | PA14 ID |  | log_2_ (Fold Change) |  | Annotation | Description |
| --- | --- | --- | --- | --- | --- | --- |
|  |  | persister VS  PA14 in LB | persister regrow 1 h VS persister | persister regrow 1.5 h VS persister regrow 1 h |  |  |
| PA2159 | PA14_36650 | 10.31 | -1.63 | -1.32 | *-* | hypothetical protein |
| PA2347 | PA14_34300 | 7.44 | -4.85 | - | *-* | DszC family monooxygenase |
| PA4139 | PA14_10380 | 7.10 | -3.36 | -1.28 | *-* | hypothetical protein |
| PA3544 | PA14_18510 | 6.94 | -3.31 | - | *algE* | alginate production outer membrane protein AlgE |
| PA2413 | PA14_33500 | 6.82 | -1.09 | - | *pvdH* | diaminobutyrate--2-oxoglutarate aminotransferase |
| PA4140 | PA14_10370 | 6.77 | -2.67 | -1.17 | *-* | hypothetical protein |
| PA0198 | PA14_02500 | 6.77 | -3.11 | - | *exbB1* | transport protein ExbB |
| PA2349 | PA14_34290 | 6.74 | -4.71 | - | *-* | DszA family monooxygenase |
| PA2171 | PA14_36520 | 6.68 | -1.09 | -1.02 | *-* | hypothetical protein |
| PA4881 | PA14_64530 | 6.67 | -1.60 | - | *-* | hypothetical protein |
| PA2424 | PA14_33280 | 6.33 | -3.51 | - | *pvdL* | peptide synthase |
| PA2355 | PA14_34200 | 6.21 | -4.02 | - | *-* | FMNH2-dependent monooxygenase |
| PA3546 | PA14_18480 | 6.17 | -3.29 | - | *algX* | alginate biosynthesis protein AlgX |
| PA3543 | PA14_18520 | 6.14 | -2.72 | - | *algK* | alginate biosynthetic protein AlgK |
| PA5482 | PA14_72370 | 5.87 | -2.85 | - | *-* | hypothetical protein |
| PA0059 | PA14_00710 | 5.86 | -1.38 | - | *osmC* | osmotically inducible protein OsmC |
| PA3442 | PA14_19580 | 5.74 | -3.57 | - | *ssuB* | aliphatic sulfonates transport ATP-binding subunit |
| PA3554 | PA14_18350 | 5.68 | -3.88 | - | *-* | bifunctional UDP-glucuronic acid decarboxylase/UDP-4-amino-4-deoxy-L-arabinose formyltransferase |
| PA2167 | PA14_36550 | 5.57 | -2.19 | -1.32 | *-* | hypothetical protein |
| PA2180 | PA14_36375 | 5.54 | -1.24 | -1.70 | *-* | hypothetical protein |
| PA2173 | PA14_36490 | 5.41 | -1.65 | - | *-* | hypothetical protein |
| PA3555 | PA14_18340 | 5.38 | -3.74 | - | *-* | hypothetical protein |
| PA2385 | PA14_33820 | 5.37 | -1.89 | - | *pvdQ* | penicillin acylase-related protein |
| PA0737 | PA14_54740 | 5.34 | -2.30 | - | *-* | hypothetical protein |
| PA0103 | PA14_01250 | 5.22 | -2.15 | - | *-* | sulfate transporter |
| PA5481 | PA14_72360 | 5.21 | -2.09 | - | *-* | hypothetical protein |
| PA3935 | PA14_12970 | 5.20 | -3.35 | - | *tauD* | taurine dioxygenase |
| PA1173 | PA14_49260 | 5.18 | -2.10 | - | *napB* | cytochrome c-type protein NapB precursor |
| PA0588 | PA14_07680 | 4.92 | -1.65 | -1.29 | *-* | hypothetical protein |
| PA0284 | PA14_03710 | 4.85 | -2.51 | - | *-* | hypothetical protein |
| PA2386 | PA14_33810 | 4.85 | -1.79 | - | *pvdA* | L-ornithine N5-oxygenase |
| PA2151 | PA14_36740 | 4.83 | -2.33 | - | *-* | hypothetical protein |
| PA0843 | PA14_53370 | 4.77 | -1.39 | - | *plcR* | phospholipase accessory protein PlcR |
| PA3062 | PA14_24500 | 4.77 | -4.68 | - | *pelC* | lipoprotein |
| PA0038 | PA14_00470 | 4.34 | -1.82 | -1.03 | *-* | hypothetical protein |
| PA0085 | PA14_01030 | 4.13 | -1.05 | - | *-* | hypothetical protein |
| PA4598 | PA14_60830 | 4.09 | -1.06 | - | *mexD* | multidrug efflux RND transporter MexD |
| PA0283 | PA14_03700 | 4.08 | -2.23 | - | *sbp* | sulfate-binding protein |
| PA4859 | PA14_64280 | 4.06 | -3.57 | - | *-* | branched-chain amino acid ABC transporter permease |
| PA0084 | PA14_01020 | 3.89 | -1.16 | -1.00 | *-* | hypothetical protein |
| PA3479 | PA14_19100 | 3.83 | -1.07 | -1.28 | *rhlA* | rhamnosyltransferase chain A |
| PA0209 | PA14_02560 | 3.75 | -2.48 | - | *-* | triphosphoribosyl-dephospho-CoA synthase |
| PA0193 | PA14_02420 | 3.67 | -1.29 | - | *-* | hypothetical protein |
| PA3333 | PA14_20950 | 3.65 | -1.05 | -1.67 | *fabH2* | 3-oxoacyl-ACP synthase |
| PA4230 | PA14_09220 | 3.65 | -1.03 | - | *pchB* | isochorismate-pyruvate lyase |
| PA4776 | PA14_63150 | 3.63 | -1.99 | - | *pmrA* | two-component response regulator |
| PA0090 | PA14_01100 | 3.59 | -1.61 | - | *-* | ClpA/B-type chaperone |
| PA2168 | PA14_36540 | 3.57 | -1.81 | - | *-* | hypothetical protein |
| PA2328 | PA14_34510 | 3.54 | -2.63 | - | *-* | hypothetical protein |
| PA3971 | PA14_12470 | 3.54 | -2.22 | - | *-* | hypothetical protein |
| PA3059 | PA14_24550 | 3.53 | -2.22 | - | *pelF* | hypothetical protein |
| PA3972 | PA14_12450 | 3.43 | -1.28 | -1.16 | *-* | acyl-CoA dehydrogenase |
| PA0195.1 | PA14_02460 | 3.42 | -1.24 | - | *-* | NAD(P) transhydrogenase subunit alpha part 2 |
| PA0096 | PA14_01170 | 3.38 | -1.58 | - | *-* | hypothetical protein |
| PA0543 | PA14_07050 | 3.38 | -1.03 | - | *-* | hypothetical protein |
| PA3335 | PA14_20920 | 3.36 | -1.04 | - | *-* | hypothetical protein |
| PA0732 | PA14_54810 | 3.35 | -1.69 | - | *-* | hypothetical protein |
| PA0091 | PA14_01110 | 3.22 | -1.41 | - | *-* | hypothetical protein |
| PA0480 | PA14_06270 | 3.18 | -1.16 | - | *-* | hydrolase |
| PA0788 | PA14_54080 | 3.17 | -1.99 | - | *-* | hypothetical protein |
| PA0083 | PA14_01010 | 3.16 | -1.42 | - | *-* | hypothetical protein |
| PA0830 | PA14_53530 | 3.11 | -2.27 | -1.05 | *-* | hypothetical protein |
| PA0624 | PA14_08100 | 3.09 | -1.12 | - | *-* | hypothetical protein |
| PA5424 | PA14_71590 | 3.08 | -1.12 | - | *-* | hypothetical protein |
| PA1210 | PA14_48650 | 3.07 | -2.06 | - | *-* | hypothetical protein |

**Table S4: The top 10 upregulated genes in the persister cells that were chosen for functional studies.**

| PAO1 ID | PA14 ID | log_2_ (Fold Change) | | | Description |
| --- | --- | --- | --- | --- | --- |
|  |  | Persister VS  PA14 in LB | Persister regrow 1 h VS persister | persister regrow 1.5 h VS persister regrow 1 h |  |
| PA2159 | PA14_36650 | 10.30 | -1.63 | -1.32 | hypothetical protein |
| PA4139 | PA14_10380 | 7.10 | -3.35 | -1.29 | hypothetical protein |
| PA4140 | PA14_10370 | 6.76 | -2.67 | -1.18 | hypothetical protein |
| PA2171 | PA14_36520 | 6.67 | -1.08 | -1.02 | hypothetical protein |
| PA4881 | PA14_64530 | 6.66 | -1.59 | - | hypothetical protein |
| PA5482 | PA14_72370 | 5.87 | -2.85 | - | hypothetical protein |
| PA2167 | PA14_36550 | 5.57 | -2.19 | -1.33 | hypothetical protein |
| PA2180 | PA14_36375 | 5.53 | -1.23 | -1.70 | hypothetical protein |
| PA2173 | PA14_36490 | 5.40 | -1.65 | - | hypothetical protein |
| PA3555 | PA14_18340 | 5.37 | -3.73 | - | hypothetical protein |

**Table S5: The potential PA2171 interacting proteins.**

| Accession | Description | MW [kDa] | Abundance |
| --- | --- | --- | --- |
| Q02GA9 | 50S ribosomal protein L21 OS=Pseudomonas aeruginosa (strain UCBPP-PA14) OX=208963 GN=rplU PE=3 SV=1 | 11.6 | 6.53E+08 |
| Q02T64 | 50S ribosomal protein L18 OS=Pseudomonas aeruginosa (strain UCBPP-PA14) OX=208963 GN=rplR PE=3 SV=1 | 12.7 | 6.44E+08 |
| Q02RL8 | 30S ribosomal protein S16 OS=Pseudomonas aeruginosa (strain UCBPP-PA14) OX=208963 GN=rpsP PE=3 SV=1 | 9.2 | 6.10E+08 |
| Q02T75 | 50S ribosomal protein L22 OS=Pseudomonas aeruginosa (strain UCBPP-PA14) OX=208963 GN=rplV PE=3 SV=1 | 11.9 | 5.37E+08 |
| Q02T81 | 30S ribosomal protein S10 OS=Pseudomonas aeruginosa (strain UCBPP-PA14) OX=208963 GN=rpsJ PE=3 SV=1 | 11.8 | 4.43E+08 |
| Q02T58 | 30S ribosomal protein S13 OS=Pseudomonas aeruginosa (strain UCBPP-PA14) OX=208963 GN=rpsM PE=3 SV=1 | 13.3 | 3.87E+08 |
| Q02T78 | 50S ribosomal protein L23 OS=Pseudomonas aeruginosa (strain UCBPP-PA14) OX=208963 GN=rplW PE=3 SV=1 | 10.9 | 3.84E+08 |
| Q02T88 | 50S ribosomal protein L7/L12 OS=Pseudomonas aeruginosa (strain UCBPP-PA14) OX=208963 GN=rplL PE=3 SV=1 | 12.5 | 3.50E+08 |
| Q02T76 | 30S ribosomal protein S19 OS=Pseudomonas aeruginosa (strain UCBPP-PA14) OX=208963 GN=rpsS PE=3 SV=1 | 10.4 | 2.95E+08 |
| A0A0H2ZGJ9 | Chorismate mutase OS=Pseudomonas aeruginosa (strain UCBPP-PA14) OX=208963 GN=pchB PE=4 SV=1 | 11.4 | 1.63E+08 |
| Q02T67 | 30S ribosomal protein S14 OS=Pseudomonas aeruginosa (strain UCBPP-PA14) OX=208963 GN=rpsN PE=3 SV=1 | 11.6 | 1.48E+08 |
| Q02GB0 | 50S ribosomal protein L27 OS=Pseudomonas aeruginosa (strain UCBPP-PA14) OX=208963 GN=rpmA PE=3 SV=1 | 9 | 1.32E+08 |
| Q02RL5 | 50S ribosomal protein L19 OS=Pseudomonas aeruginosa (strain UCBPP-PA14) OX=208963 GN=rplS PE=3 SV=1 | 13 | 1.23E+08 |
| Q02T71 | 30S ribosomal protein S17 OS=Pseudomonas aeruginosa (strain UCBPP-PA14) OX=208963 GN=rpsQ PE=3 SV=1 | 10.1 | 9.38E+07 |
| Q02T66 | 30S ribosomal protein S8 OS=Pseudomonas aeruginosa (strain UCBPP-PA14) OX=208963 GN=rpsH PE=3 SV=1 | 14.2 | 9.12E+07 |
| Q02T57 | 30S ribosomal protein S11 OS=Pseudomonas aeruginosa (strain UCBPP-PA14) OX=208963 GN=rpsK PE=3 SV=1 | 13.6 | 8.61E+07 |
| Q02T70 | 50S ribosomal protein L14 OS=Pseudomonas aeruginosa (strain UCBPP-PA14) OX=208963 GN=rplN PE=3 SV=1 | 13.4 | 8.16E+07 |
| A0A0H2ZFW8 | Pilin OS=Pseudomonas aeruginosa (strain UCBPP-PA14) OX=208963 GN=pilA PE=3 SV=1 | 18.1 | 6.35E+07 |
| Q02F84 | 30S ribosomal protein S18 OS=Pseudomonas aeruginosa (strain UCBPP-PA14) OX=208963 GN=rpsR PE=3 SV=1 | 8.9 | 6.34E+07 |
| Q02FT1 | 30S ribosomal protein S15 OS=Pseudomonas aeruginosa (strain UCBPP-PA14) OX=208963 GN=rpsO PE=3 SV=1 | 10.1 | 5.90E+07 |
| Q02T59 | 50S ribosomal protein L36 1 OS=Pseudomonas aeruginosa (strain UCBPP-PA14) OX=208963 GN=rpmJ1 PE=3 SV=1 | 4.4 | 5.58E+07 |
| Q02T65 | 50S ribosomal protein L6 OS=Pseudomonas aeruginosa (strain UCBPP-PA14) OX=208963 GN=rplF PE=3 SV=1 | 19.1 | 5.46E+07 |
| A0A0H2ZHL2 | Uncharacterized protein OS=Pseudomonas aeruginosa (strain UCBPP-PA14) OX=208963 GN=PA14_62240 PE=4 SV=1 | 12.9 | 5.35E+07 |
| Q02T61 | 50S ribosomal protein L15 OS=Pseudomonas aeruginosa (strain UCBPP-PA14) OX=208963 GN=rplO PE=3 SV=1 | 15.2 | 5.17E+07 |
| Q02E46 | 50S ribosomal protein L28 OS=Pseudomonas aeruginosa (strain UCBPP-PA14) OX=208963 GN=rpmB PE=3 SV=1 | 9.1 | 4.25E+07 |
| Q02DF5 | ATP synthase epsilon chain OS=Pseudomonas aeruginosa (strain UCBPP-PA14) OX=208963 GN=atpC PE=3 SV=1 | 14.7 | 4.01E+07 |
| Q02GB4 | 30S ribosomal protein S20 OS=Pseudomonas aeruginosa (strain UCBPP-PA14) OX=208963 GN=rpsT PE=3 SV=1 | 9.9 | 3.72E+07 |
| Q02DE9 | ATP synthase subunit c OS=Pseudomonas aeruginosa (strain UCBPP-PA14) OX=208963 GN=atpE PE=3 SV=1 | 8.6 | 3.45E+07 |
| Q02EW8 | 50S ribosomal protein L31 OS=Pseudomonas aeruginosa (strain UCBPP-PA14) OX=208963 GN=rpmE PE=1 SV=1 | 7.9 | 2.73E+07 |
| Q02T54 | 50S ribosomal protein L17 OS=Pseudomonas aeruginosa (strain UCBPP-PA14) OX=208963 GN=rplQ PE=3 SV=1 | 14.5 | 2.64E+07 |
| Q02T82 | Elongation factor Tu OS=Pseudomonas aeruginosa (strain UCBPP-PA14) OX=208963 GN=tuf1 PE=1 SV=1 | 43.3 | 7.42E+08 |
| Q02DF2 | ATP synthase subunit alpha OS=Pseudomonas aeruginosa (strain UCBPP-PA14) OX=208963 GN=atpA PE=3 SV=1 | 55.4 | 3.40E+08 |
| A0A0H2Z855 | Ribonucleoside-diphosphate reductase OS=Pseudomonas aeruginosa (strain UCBPP-PA14) OX=208963 GN=nrdA PE=3 SV=1 | 107 | 3.35E+08 |
| A0A0H2ZCA6 | DNA gyrase subunit A OS=Pseudomonas aeruginosa (strain UCBPP-PA14) OX=208963 GN=gyrA PE=3 SV=1 | 100.9 | 2.99E+08 |
| Q02KU3 | Trigger factor OS=Pseudomonas aeruginosa (strain UCBPP-PA14) OX=208963 GN=tig PE=3 SV=1 | 48.6 | 2.68E+08 |
| Q02HI0 | Alkaline phosphatase L OS=Pseudomonas aeruginosa (strain UCBPP-PA14) OX=208963 GN=phoA2 PE=2 SV=1 | 40.7 | 2.40E+08 |
| A0A0H2ZI23 | Transcription termination/antitermination protein NusA OS=Pseudomonas aeruginosa (strain UCBPP-PA14) OX=208963 GN=nusA PE=3 SV=1 | 54.6 | 2.03E+08 |
| Q02T55 | DNA-directed RNA polymerase subunit alpha OS=Pseudomonas aeruginosa (strain UCBPP-PA14) OX=208963 GN=rpoA PE=3 SV=1 | 36.6 | 1.99E+08 |
| A0A0H2ZJB9 | D-amino acid dehydrogenase OS=Pseudomonas aeruginosa (strain UCBPP-PA14) OX=208963 GN=dadA PE=3 SV=1 | 47.1 | 1.84E+08 |
| A0A0H2ZIL4 | Pyruvate dehydrogenase E1 component OS=Pseudomonas aeruginosa (strain UCBPP-PA14) OX=208963 GN=aceA PE=4 SV=1 | 99.5 | 1.81E+08 |
| A0A0H2Z7Z1 | Ribonucleoside-diphosphate reductase subunit beta OS=Pseudomonas aeruginosa (strain UCBPP-PA14) OX=208963 GN=nrdB PE=3 SV=1 | 47.4 | 1.54E+08 |
| A0A0H2Z8N2 | Aconitate hydratase B OS=Pseudomonas aeruginosa (strain UCBPP-PA14) OX=208963 GN=acnB PE=3 SV=1 | 93.6 | 1.51E+08 |
| A0A0H2ZID8 | Acetyltransferase component of pyruvate dehydrogenase complex OS=Pseudomonas aeruginosa (strain UCBPP-PA14) OX=208963 GN=aceF PE=3 SV=1 | 56.7 | 1.31E+08 |
| A0A0H2ZDD5 | 30S ribosomal protein S1 OS=Pseudomonas aeruginosa (strain UCBPP-PA14) OX=208963 GN=rpsA PE=3 SV=1 | 61.8 | 1.13E+08 |
| A0A0H2ZEE2 | Glycerol-3-phosphate dehydrogenase OS=Pseudomonas aeruginosa (strain UCBPP-PA14) OX=208963 GN=glpD PE=3 SV=1 | 57.1 | 1.11E+08 |
| Q02EW3 | ATP-dependent protease ATPase subunit HslU OS=Pseudomonas aeruginosa (strain UCBPP-PA14) OX=208963 GN=hslU PE=3 SV=1 | 50.1 | 1.01E+08 |
| A0A0H2ZF77 | Outer membrane protein assembly factor BamB OS=Pseudomonas aeruginosa (strain UCBPP-PA14) OX=208963 GN=bamB PE=3 SV=1 | 40.4 | 9.32E+07 |
| A0A0H2ZA71 | PvdN OS=Pseudomonas aeruginosa (strain UCBPP-PA14) OX=208963 GN=pvdN PE=3 SV=1 | 47.9 | 8.91E+07 |
| A0A0H2ZAX9 | Isocitrate lyase OS=Pseudomonas aeruginosa (strain UCBPP-PA14) OX=208963 GN=aceA PE=4 SV=1 | 58.8 | 8.15E+07 |
| Q02NB5 | Isocitrate dehydrogenase [NADP] OS=Pseudomonas aeruginosa (strain UCBPP-PA14) OX=208963 GN=icd PE=1 SV=1 | 45.5 | 8.00E+07 |
| A0A0H2ZHT6 | Carbamoyl-phosphate synthase large chain OS=Pseudomonas aeruginosa (strain UCBPP-PA14) OX=208963 GN=carB PE=3 SV=1 | 117.3 | 7.88E+07 |
| Q02PB3 | Enoyl-[acyl-carrier-protein] reductase [NADH] OS=Pseudomonas aeruginosa (strain UCBPP-PA14) OX=208963 GN=fabV PE=3 SV=1 | 43.5 | 7.36E+07 |
| A0A0H2ZHB4 | Chaperone protein ClpB OS=Pseudomonas aeruginosa (strain UCBPP-PA14) OX=208963 GN=clpB PE=3 SV=1 | 94.9 | 7.28E+07 |
| A0A0H2ZAX4 | Adenylosuccinate lyase OS=Pseudomonas aeruginosa (strain UCBPP-PA14) OX=208963 GN=purB PE=3 SV=1 | 50.5 | 6.53E+07 |
| Q02H55 | Chaperonin GroEL OS=Pseudomonas aeruginosa (strain UCBPP-PA14) OX=208963 GN=groEL PE=3 SV=1 | 57.1 | 6.24E+07 |
| A0A0H2ZKS8 | Anaerobically-induced outer membrane porin OprE OS=Pseudomonas aeruginosa (strain UCBPP-PA14) OX=208963 GN=oprE PE=3 SV=1 | 49.8 | 5.94E+07 |
| Q02K73 | Succinate--CoA ligase [ADP-forming] subunit beta OS=Pseudomonas aeruginosa (strain UCBPP-PA14) OX=208963 GN=sucC PE=3 SV=1 | 41.5 | 5.82E+07 |
| A0A0H2ZEU1 | RNA polymerase sigma factor RpoS OS=Pseudomonas aeruginosa (strain UCBPP-PA14) OX=208963 GN=rpoS PE=3 SV=1 | 38.2 | 5.81E+07 |
| A0A0H2ZM25 | Cell division protein FtsZ OS=Pseudomonas aeruginosa (strain UCBPP-PA14) OX=208963 GN=ftsZ PE=3 SV=1 | 41.1 | 5.70E+07 |
| A0A0H2ZF14 | Valine--tRNA ligase OS=Pseudomonas aeruginosa (strain UCBPP-PA14) OX=208963 GN=valS PE=3 SV=1 | 107.7 | 5.70E+07 |

**Table S6: Changes in the expression levels of genes related to oxidative stress, SOS response, stringent response in the RNA-seq results.**

| PAO1 ID | PA14 ID | log_2_ (Fold Change)  persister VS PA14 in LB | Annotation | Description |
| --- | --- | --- | --- | --- |
| PA0139 | PA14_01710 | 1.63 | *ahpC* | alkyl hydroperoxide reductase subunit C |
| PA0140 | PA14_01720 | 2.90 | *ahpF* | alkyl hydroperoxide reductase subunit F |
| PA4236 | PA14_09150 | 3.13 | *katA* | catalase KatA |
| PA4613 | PA14_61040 | 5.12 | *katB* | catalase KatB |
| PA0003 | PA14_00030 | -1.86 | *recF* | DNA replication/repair protein RecF |
| PA4763 | PA14_63010 | 1.02 | *recN* | DNA repair protein RecN |
| PA4468 | PA14_58000 | *-* | *sodA* | - |
| PA4366 | PA14_56780 | 1.30 | *sodB* | superoxide dismutase [Fe] |
| PA0934 | PA14_52180 | *-* | *relA* | - |
| PA5338 | PA14_70470 | -1.55 | *spoT* | bifunctional GTP diphosphokinase/guanosine-3'%2C5'-bis pyrophosphate 3'-pyrophosphohydrolase |
| PA2585 | PA14_30660 | -1.64 | *uvrC* | excinuclease ABC subunit UvrC |
| PA5443 | PA14_71870 | *-* | *uvrD* | - |

**
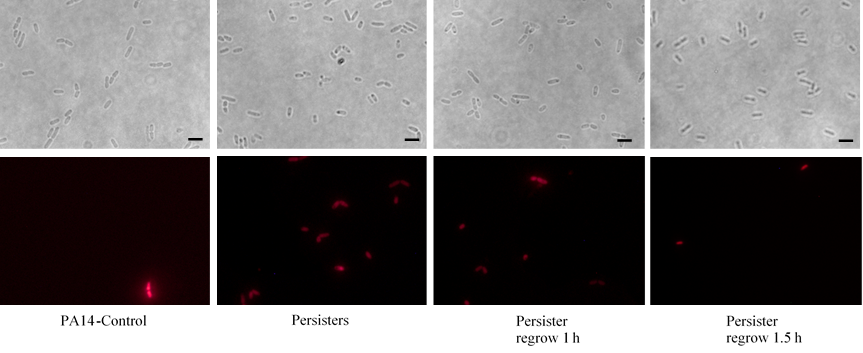
**

**Fig. S1** ropidium iodide (PI) staining of collected cells collected at indicated time points. Scale bar, 3 μm.


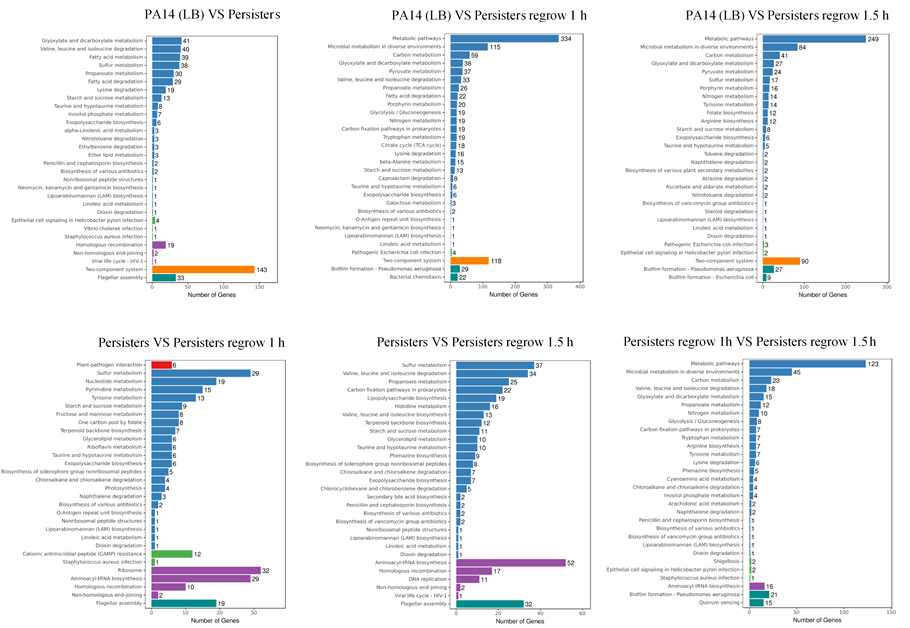


(e)

(a)

(b)

(c)

(d)

(f)

**Fig. S2.** Statistical chart of differential gene pathway distribution. The vertical axis represents pathway names, the horizontal axis represents the number of genes, and different colors indicate different pathway categories.


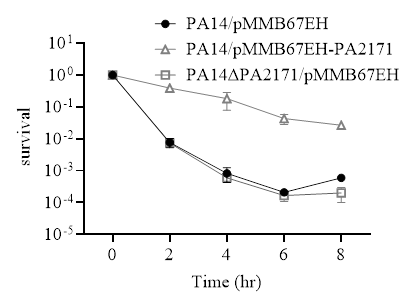


**Fig. S3** The survival curves of wile type PA14 with pMMB67EH empty vector, overexpressing pMMB67EH-PA2171 and wile type PA14 with PA2171 deletion and pMMB67EH empty vector following meropenem (8 μg/mL) treatment.


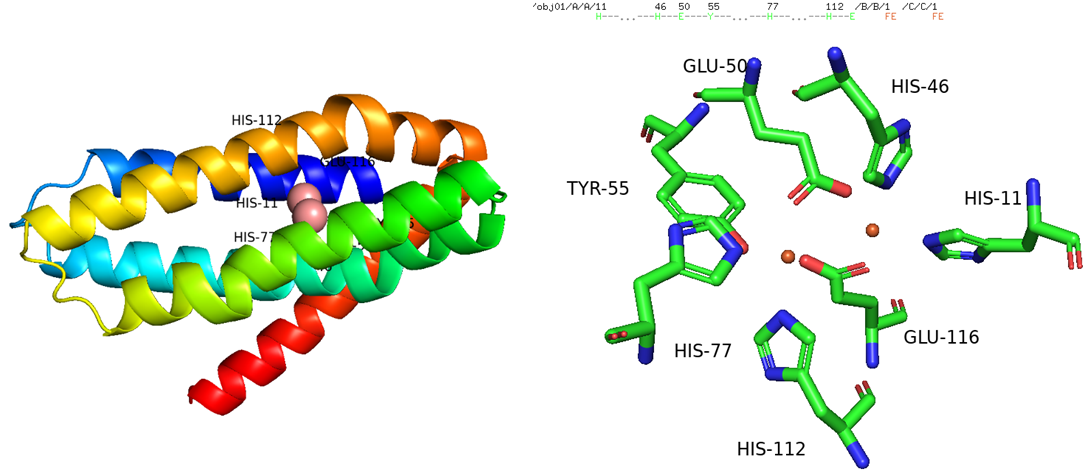


**Fig. S4** The protein structure of PA2171 predicted by AlphaFold3, each PA2171 monomer can bind two divalent cations.


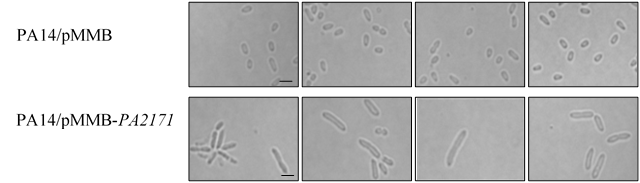


**Fig. S5** Overexpression of *PA2171* in PA14 affects the cell length. Scale bar, 3 μm.


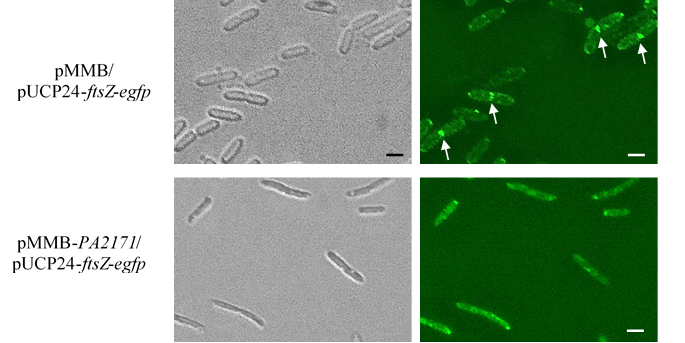


**Fig. S6** Observation of FtsZ ring formation under the fluorescence microscope. The FtsZ rings at the cell’s centers were indicated by arrows. Scale bar, 1 μm.
